# Outcome and Treatment Effects in Stroke Associated with Acute Cervical ICA Occlusion

**DOI:** 10.1371/journal.pone.0170247

**Published:** 2017-01-12

**Authors:** Michael Gliem, John-Ih Lee, Aurica Barckhan, Bernd Turowski, Hans-Peter Hartung, Sebastian Jander

**Affiliations:** 1 Department of Neurology, Heinrich-Heine-University, Medical Faculty, Düsseldorf, Germany; 2 Department of Diagnostic and Interventional Radiology, Heinrich-Heine-University, Medical Faculty, Düsseldorf, Germany; University Hospital-Eppendorf, GERMANY

## Abstract

**Background:**

Endovascular therapy (EVT) with stent retrievers in addition to i.v. thrombolysis (IVT) has proven effective in acute stroke patients with middle cerebral artery (MCA, M1 segment) and distal internal carotid artery (ICA) occlusion. Limited data exist concerning acute cervical ICA occlusion, either alone or in combination with intracranial ICA occlusion (tandem occlusion). Therefore we analyzed outcome and treatment effects in stroke associated with cervical ICA occlusion, with specific focus on the impact of intracranial ICA or M1 patency.

**Methods:**

Seventy-eight patients with cervical ICA occlusion from our local stroke unit registry were analyzed retrospectively. Thrombolysis in Cerebral Infarction (TICI) classification, infarct size, modified Rankin scale (mRS), symptomatic intracerebral hemorrhage (ICH), and death were assessed as outcome parameters.

**Results:**

Forty-three patients had isolated cervical ICA occlusion whereas 35 patients presented with extra-/intracranial tandem occlusion. Patients underwent IVT alone (n = 23), combined IVT/EVT (n = 28) or no treatment (n = 27). Treated and untreated patients with tandem occlusion had a worse outcome after 90 days compared to isolated cervical occlusion (OR for moderate outcome 0.29, 0.27–0.88, p = 0.01). Additional EVT improved outcome in patients with tandem occlusion (OR for moderate outcome: 15.43, 1.60–148.90, p = 0.008) but not isolated cervical occlusion (OR 1.33, 0.38–11.60, NS).

**Conclusions:**

In contrast to tandem occlusion, stroke outcome in patients with isolated cervical ICA occlusion was generally more benign and not improved by combined IVT/EVT compared to IVT alone. Intracranial vessel patency may be critical for treatment decision in acute cervical ICA occlusion.

## Introduction

Systemic i.v. thrombolysis (IVT) with rt-PA is the standard treatment for ischemic stroke in the 4.5 hour time window [1]. Recently, endovascular treatment (EVT) with stent retrievers in addition to IVT has shown significant benefit with regard to recanalization rates and clinical outcome in patients with intracranial large-vessel occlusion, i.e. M1, carotid T or intracranial internal carotid artery (ICA) occlusion [2–6]. However, the treatment strategy for cervical ICA occlusion is less clear. The ICARO-3 trial showed a similar outcome after IVT and EVT in stroke associated with cervical ICA occlusion, but did not specifically assess the status of M1 or carotid T perfusion [7]. We therefore addressed outcome and treatment effects in stroke associated with cervical ICA occlusion, with specific focus on the impact of intracranial ICA or M1 patency.

## Methods

### Study population

We hypothesized that benefit to recanalization therapy of acute cervical ICA occlusion depends on intracranial vessel patency. We therefore compared outcome of IVT and EVT in cervical ICA occlusion with or without additional MCA occlusion. Routine medical care data collected for quality control measures of all patients treated for ischemic stroke in the Stroke unit of the University Hospital Düsseldorf were analyzed retrospectively in an anonymized and pseudonymized manner. The study was approved by the Ethikkommission an der Med. Fakultät der HHU Düsseldorf (#4743R). For observational retrospective analysis of anonymized and pseudonymized routine care data a separate written informed consent was not required by the local ethics committee.

From 12/2010–06/2014 we identified 78 patients with ischemic stroke and documented occlusion of the cervical ICA in ultrasound, magnetic resonance (MR) angiography, computed tomographic (CT) angiography or digital substraction angiography within 24 hours after admission. In ultrasound occlusion was characterized by absence of any signal along the extracranial course of the ICA and a low-velocity signal and absent diastolic flow at the origin of the ICA. ICA occlusion was defined as no flow at all in DSA or TOF-MRA in the ICA course between carotid bifurcation and branching of ophthalmic artery often accompanied by elevated flow in collaterals. In CTA occlusion was defined as lack of any contrast-enhancement based on visual analysis and ROI density measurements in the course of the ICA.

Thirty-five patients with ischemic stroke and documented occlusion of the cervical ICA had an additional occlusion of the proximal middle cerebral artery (MCA, M1 segment) or the carotid-T, i.e. tandem occlusion. Recanalization treatment was considered in patients with NIHSS >4 or with a functionally relevant functional deficit. Patients were treated with either IVT or with IVT followed by EVT (IVT/EVT) based on interdisciplinary decision and availability of the neurointerventionalist. If additional EVT was indicated, patients were immediately transferred to the angiography room under ongoing IVT without awaiting the response to IVT. ICA occlusion was treated with Wallstent and PTA in 20 patients, and stent retrievers in eight. Intracranial vessel occlusions were treated with stent-retrievers in 15 of 16 patients. Patients with contraindication against IVT but receiving EVT were excluded. Patients not eligible for any treatment were also analyzed.

Sociodemographic data, stroke risk factors and periprocedural data like NIHSS, onset-to-needle time, and recanalization rate were documented. Five patients were excluded due to missing mRS90 data.

### Diagnostic tools

Imaging was performed with a 3-T or 1.5-T MR scanner (MRT: T2*, DWI, ADC, FLAIR, TOF) or contrast enhanced CT (5 mm slices, 0.75 mm slices for CT-angiography).

### Outcome assessment

MCA recanalization was assessed using the TICI score [8]. A TICI score with values of 2b or 3 in a post treatment CT or MR angiography or in the last digital substraction angiography series was considered to indicate successful recanalization. Cervical ICA recanalization was assessed by extracranial duplex sonography. Infarct size in follow-up imaging by MR or CT was documented as either > 1/3 or < 1/3 MCA territory. ECASS criteria [9] were used to define secondary bleeding complications. Clinical outcome was assessed using modified Ranking Scale (mRS). Outcome in mRS after 3 months was assessed by a standardized telephone interview. mRS ≤ 2 was considered favorable, mRS ≤ 3 was considered moderate and mRS > 3 was considered poor outcome. Mortality was assessed within 90 days after treatment.

### Statistical analysis

SPSS (IBM, Armonk, NY) and GraphPad Prism^™^ software (GraphPad Software Inc., La Jolla, CA) were used for statistical analysis. Treatment groups and dichotomized parameters were compared using the Fisher-Exact Test. Continuous data were analyzed with the Mann-Whitney *U* test. All tests were two tailed and results were assumed statistically significant with a p < 0.05.

## Results

### Overall stroke severity and outcome

Out of 78 acute stroke patients with cervical ICA occlusion, 35 had an additional MCA occlusion, i.e. extra-/intracranial tandem occlusion. This was associated with increased stroke severity (Median NIHSS 16 vs 8, Mann-Whitney U test, p<0.001) and worse outcome after 90 days (OR for moderate outcome 0.29, 0.27–0.88, p = 0.01).

### Treatment effects

Emergency recanalization was performed in 51 patients whereas 27 patients did not qualify for treatment due to delayed admission or low NIHSS.

**Fig 1:**
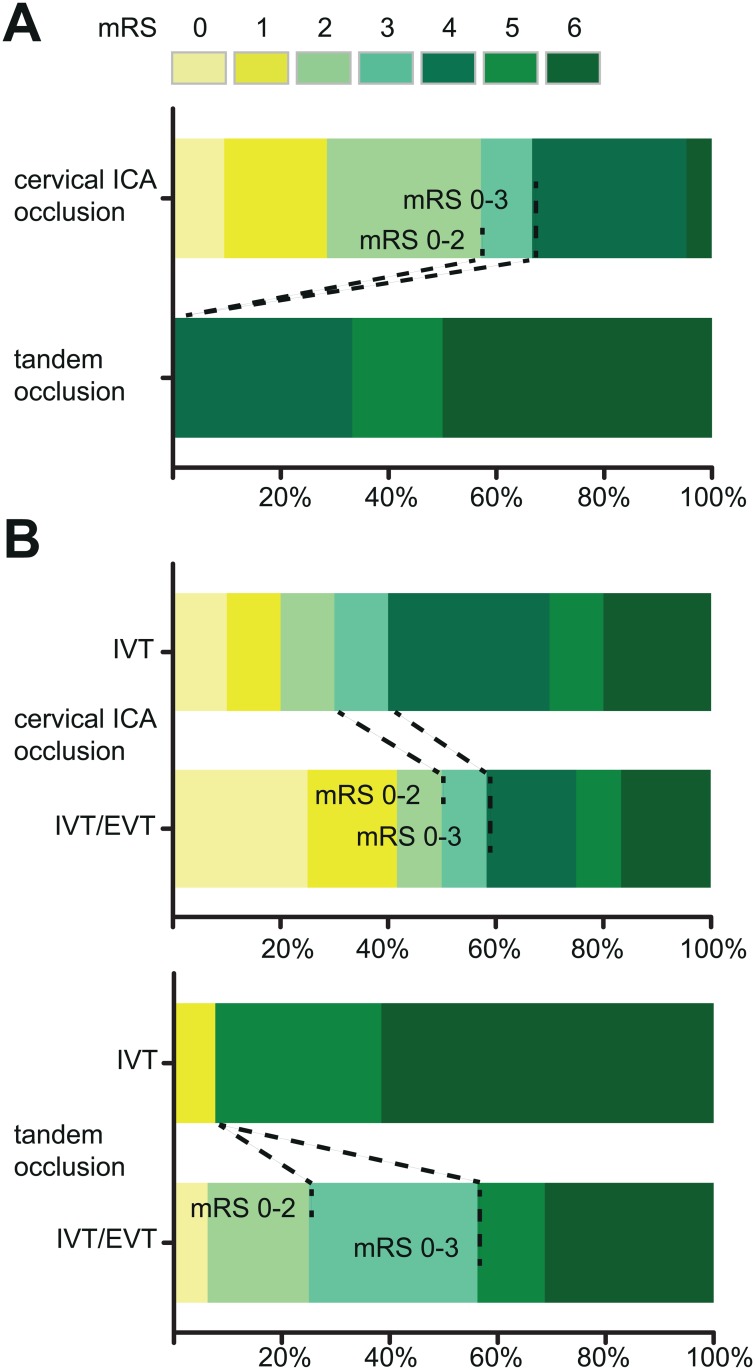
Outcome and treatment effects in patients with isolated cervical ICA occlusion and extra-/intracranial tandem occlusion. (A) Outcome according to modified Rankin scale (mRS) scores at day 90 in untreated patients with isolated cervical ICA occlusion and extra-/intracranial tandem occlusion, respectively. Scores range from 0–6, 0 indicating no symptoms, 1 no clinically significant disability despite symptoms, 2 slight disability (ability to look after own affairs, but unable to perform all previous activities), 3 moderate disability (some help needed but able to walk unassisted), 4 moderately severe disability (unability to walk alone and care for bodily needs without assistance), 5 severe disability (constant nursing care and attention required), 6 death. Compared to patients with tandem occlusion, favorable outcome (mRS 0–2) and moderate outcome (mRS 0–3) occurred significantly more often in the isolated cervical ICA occlusion group (mRS 0–2: Fisher’s exact test p = 0.02, mRS 0–3: Fisher’s exact test p = 0.006). (B) Effect of combined intravenous/endovascular treatment (IVT/EVT) compared to i.v. treatment alone (IVT). In patients with isolated cervical ICA occlusion, outcome was not significantly different between the IVT/EVT and IVT group (mRS 0–3, Fisher’s exact test p = 0.67). In tandem occlusion, however, we found an increased rate of moderate outcome in IVT/EVT-treated patients compared to IVT alone (Fisher’s exact test p = 0.008). Shift analysis over the whole range of mRS confirmed this finding (Mann-Whitney U p = 0.02). See (A) for details of mRS scoring.

In untreated patients, moderate outcome (mRS 0–3) was reached by 14/21 patients with isolated ICA occlusion, but 0/6 of patients with extra-/intracranial tandem occlusion (Fisher’s exact test p = 0.006) (Fig 1A).

In patients with tandem occlusion combined IVT/EVT showed a trend towards an increased recanalization rate and reduced infarct size compared to IVT alone (Table 1). Moderate outcome (IVT/EVT 9/16 vs. IVT 1/13, OR 15.43, 1.60–148.90, Fisher’s exact test p = 0.008) but not favorable outcome (IVT/EVT 4/16 vs. IVT 1/13, OR 4, 0.38–41.25, p = 0.34; Fig 1B, Table 1) was increased. Shift analysis over the whole range of mRS revealed significant improvement of IVT/EVT-treated patients with tandem occlusion (Mann-Whitney U test, p = 0.02).

**Table 1:** Treatment effects in isolated cervical ICA occlusion and in ICA-MCA tandem occlusion, respectively.

**Isolated cervical ICA occlusion**					
**Outcome**	**IVT (n = 10)**	**IVT/EVT (n = 12)**	**P Value**	**OR**	**95% CI**
**Successful recanalization ICA**	0	11	**<0.0001**	**161**	**5.89–4404**
**Infarct size <1/3 MCA**	5	7	1	1.4	0.26–7.59
**sICH**	0	3	0.22	7.73	0.35–170.20
**Mortality**	2	2	1	0.67	0.08–5.75
**Favourable outcome (mRS 0–2)**	3	6	0.41	2.33	0.39–13.62
**Moderate outcome (mRS 0–3)**	4	7	0.67	1.33	0.38–11.60
**ICA-MCA tandem occlusion**					
**Outcome**	**IVT (n = 13)**	**IVT/EVT (n = 16)**	**P Value**	**OR**	**95% CI**
**Successful recanalization MCA**	3	10	0.06	5.6	0.94–7.8
**Infarct size <1/3 MCA**	1	8	**0.02**	**12.00**	**1.25–115.4**
**sICH**	1	5	0.18	5.45	0.54–54.31
**Mortality**	8	5	0.14	0.28	0.06–1.32
**Favourable outcome (mRS 0–2)**	1	4	0.34	4	0.38–41.25
**Moderate outcome (mRS 0–3)**	1	9	**0.008**	**15.43**	**1.60–148.90**

In patients with isolated cervical ICA occlusion recanalization was substantially increased upon IVT/EVT (11/12, vs 0/10 after IVT alone, p<0.0001). However, although there was a trend toward better outcome of EVT-treated patients with isolated cervical occlusion, the effect was much smaller than in the tandem occlusion group and not significant in statistical analysis (favorable outcome: IVT/EVT 6/12, IVT 3/10, OR 2.33, 0.39–13.62, Fisher’s exact test p = 0.41; moderate outcome: IVT/EVT 7/12, IVT 4/10, OR 1.33, 0.38–11.60, p = 0.67; Fig 1B). Shift analysis confirmed similar efficacy of IVT and combined IVT/EVT in the cervical occlusion group (Mann-Whitney U test, p = 0.49).

Apart from NIHSS, baseline characteristics in the group of untreated patients with ICA occlusion or combined ICA-MCA occlusion were balanced (Table 2). Etiology of ICA occlusion was not different between the groups (S1 Table).

**Table 2:** Baseline characteristics of untreated patients with isolated ICA occlusion and ICA-MCA tandem occlusion.

Characteristics	Isolated ICA occlusion without IVT/EVT (n = 21)	ICA-MCA occlusion without IVT/EVT (n = 6)	P Value
**Male sex**	13 (62%)	4 (67%)	1.000
**Median age (y)**	67 (56–75)	62 (52–86)	0.838
**Median NIHSS Score**	4 (3–8.5)	16.5 (11.75–21.25)	**0.003**
**Arterial hypertension**	20 (95%)	5 (83%)	0.402
**Diabetes mellitus**	7 (33%)	3 (50%)	0.638
**Atrial fibrillation**	4 (19%)	1 (17%)	1.000
**Hyperlipidemia**	16 (76%)	2 (33%)	0.136
**Smoking**	10 (48%)	1 (17%)	0.350
**Previous stroke**	9 (43%)	1 (17%)	0.363
**Coronary heart disease**	4 (19%)	1 (17%)	1.000

In treated patients with isolated ICA occlusion baseline characteristics were similar in IVT- and IVT/EVT-treated groups (Table 3). Etiology of ICA occlusion was not different and was classified as macroangiopathy, most likely of atherosclerotic origin, in 4/10 IVT treated and 4/12 IVT/EVT treated patients. In overall 7 Patients another determined etiology according to TOAST criteria was documented, of which 6 patients had a dissection. We found 1 dissection in the ICA-IVT group and 2 dissections in the ICA-IVT/EVT group (S1 Table). In the tandem occlusion group IVT/EVT-treated patients were younger than those treated by IVT alone (Table 3) but age was not correlated with treatment effects, in line with data from IST-3 [10] and recent thrombectomy trials (MR CLEAN [2], ESCAPE [4]). Etiology of ICA occlusion was not different and classified as macroangiopathy in 4/13 tandem occlusions treated by IVT and 7/16 tandem occlusions treated by IVT/EVT. Dissections were not detected in treated patients with tandem occlusion (S1 Table).

**Table 3:** Baseline characteristics of treated patients with isolated ICA occlusion and ICA-MCA tandem occlusion.

Characteristics	Isolated ICA occlusion IVT (n = 10)	Isolated ICA occlusion IVT/EVT (n = 12)	P Value	ICA-MCA Occlusion IVT (n = 13)	ICA-MCA Occlusion IVT/EVT (n = 16)	P Value
**Male sex**	5 (50%)	8 (67%)	0.666	5 (39%)	7 (44%)	1.000
**Median age (y)**	76 (70–84)	64 (57–76)	0.111	**86 (76–91)**	**72 (57–77)**	**0.004**
**Median NIHSS Score**	13 (8–18)	12 (5–17)	0.792	17 (15–19)	15 (11–17)	0.17
**Arterial hypertension**	10 (100%)	9 (75%)	0.221	11 (85%)	12 (75%)	0.663
**Diabetes mellitus**	2 (20%)	3 (25%)	1.0	1 (8%)	1 (6%)	1.0
**Atrial fibrillation**	4 (40%)	3 (25%)	0.652	9 (69%)	5 (31%)	0.066
**Hyperlipidemia**	5 (50%)	7 (58%)	1.0	8 (61%)	8 (50%)	0.711
**Smoking**	1 (10%)	4 (33%)	0.323	2 (15%)	3 (19%)	1.000
**Previous stroke**	3 (30%)	2 (17%)	0.624	3 (23%)	0 (0%)	0.078
**Coronary heart disease**	1 (10%)	1 (8%)	1.0	4 (31%)	1 (6%)	0.144
**Site of MCA occlusion**						
**M1 (main stem)**	0	0	-	9(69%)	8(50%)	0.451
**M2/M3**	2 (20%)	2 (17%)	1.0	0	0	-
**Carotid T**	0	0	-	4 (31%)	8(50%)	0.451
**Median onset to IV rTPA (min)**	88 (74–236)	80 (64–149)	0.390	115 (96–129),	89 (47–144)	0.255
**Median onset to endovascular treatment (min)**	Not applicable	162 (128–264)	-	Not applicable	177 (125–250)	**-**

## Discussion

The recent randomized trials showed significant benefit of EVT in stroke due to intracranial large artery occlusion [2–6]. In the clinical setting, however, many patients present with clinical characteristics placing them outside the trial inclusion criteria, among them patients with isolated cervical ICA occlusion, raising the question if these patients should be treated even in case of patent intracranial MCA and/or distal ICA. So far, optimum treatment strategies are ill-defined.

Our study shows that outcome after stroke in patients with acute cervical ICA occlusion critically depends on intracranial vessel patency and is relatively benign in patients with isolated cervical occlusion. Regarding treatment effects, we found a significant benefit of EVT in extra-/intracranial tandem occlusion but a much smaller effect in isolated cervical occlusion which was not significant in our limited sample size. Therefore, intracranial vessel patency may be critical for the treatment decision in acute cervical ICA occlusion.

In contrast to our data, a systematic review of previous trials concluded that outcome in patients with isolated ICA occlusion but not tandem occlusion would be improved by EVT [11]. However, the studies included into this meta-analysis comprised very heterogeneous regimens of EVT, due to the fact that they were conducted over a long period of nearly 20 years. In contrast 93% of our patients with tandem occlusion were treated with a stent retriever, which may explain the improved outcome in tandem occlusion in our study compared to previous results.

The multicenter ICARO-3 [7] study on the other hand compared IVT and EVT in cervical artery occlusion and found that the clinical outcome after 90 days was similar after IVT and EVT. However, intracranial vessel status was not systematically addressed in this study. Therefore, conclusions regarding the therapeutic outcome in isolated cervical ICA occlusions relative to tandem occlusion cannot be drawn from this trial.

In the recently published thrombectomy trials patients with isolated cervical ICA occlusion were not included. Regarding extra-/intracranial tandem occlusion a prespecified subgroup analysis of the MR CLEAN [2] trial showed that an additional cervical ICA occlusion (148 of 500 included patients) reduced the OR favoring EVT from 1.85 (1.26–2.72) to 1.43 (0.78–2.64), corresponding to a non-significant result in the tandem occlusion subgroup. In the REVASCAT [6] trial ICA/MCA tandem occlusion (55 patients) was also analyzed and, in contrast to the MR CLEAN data, showed significant improvement after endovascular treatment not significantly different from isolated intracranial occlusion. Similar results were reported in the ESCAPE [4] and SWIFT PRIME [5] (30 patients) trials. Similar to the large randomized trials recent observational registry studies excluded patients with isolated cervical ICA occlusion [12,13]. Therefore our study presents the first data addressing clinical outcome in this patient group.

We were not able to demonstrate an increased rate of favorable outcome (mRS ≤2) in any treatment group. However, patients presenting with stroke due to ICA-MCA tandem occlusion are severely affected reflected by high NIHSS and mRS scores. mRS 0–3, referred to as moderate outcome, is an accepted limit of successful treatment in severely affected patients [14]. Using these limits, we could detect an increased rate of moderate outcome upon IVT/EVT of tandem occlusion patients which could be confirmed by shift analysis for the detection of changes over the whole range of mRS.

Limitations of our study include retrospective analysis of routine care data with lack of randomization and the small sample size, potentially precluding the detection of smaller beneficial effects of EVT in patients with isolated cervical ICA occlusion. Therefore larger randomized trials of EVT in isolated cervical ICA occlusion are necessary.

## Supporting Information

S1 Table Data underlying the analyses. (PDF)
