# Supplementary material for: Outcome and Treatment Effects in Stroke Associated with Acute Cervical ICA Occlusion
Source: PLoS One. 2017 Jan 12;12(1):e0170247. doi: 10.1371/journal.pone.0170247 (PMC5231377; doi:10.1371/journal.pone.0170247)
Supplement: S1 Table — (PDF) [file pone.0170247.s001.pdf]

PATNO	GENDER	AGE	NIHSS	HYPERTON 1=no/2=yes	DIABETES 1=no/2=yes	AF 1=no/2=yes	HLP 1=no/2=yes	SMOKE 1=no/2=yes	PRIOR STROKE 1=no/2=yes	KHK 1=no/2=yes	ONSET-TO-DOOR 1=<3hr 2=3-4,5hr 3=4.5-6hr 4=6-24hr 5=>24hr or unknown	SITE OF OCCLUSION	ONTOIVT minutes	ONSET TO EVT minutes	RECANAL TICI 5=unknown	INFARCT SIZE	ICH	SYMPTOMATIC ICH	mRS90	TOAST 1=atherosclerotic 2=cardioembolic 3=small vessel 4=other determined etiology 5=undetermined etiology	OCCLUSION DETECTED BY	ECD	TCD	CTA	MRA
**ICA occlusion without IVT/EVT (21 Pat)**																									
1	2	87	21	2	1	1	1	1	1	1	4	ICA			0	> 1/3 MCA	no		6	5		X		X	
2	1	57	3	2	1	1	2	2	2	1	1	ICA			0	< 1/3 MCA	no		2	1		X			X
3	2	75	5	2	2	1	2	1	1	1	4	ICA			5	< 1/3 MCA	no		2	1		X			X
4	1	62	4	2	1	1	2	1	2	2	5	ICA			5	< 1/3 MCA	no		1	1		X		X	
5	1	54	3	2	1	1	2	2	1	1	5	ICA			5	< 1/3 MCA	no		1	1				X	
6	1	73	0	2	2	1	2	1	2	2	4	ICA			0	<1/3 MCA	no		2	1		X		X	X
7	2	85	5	2	2	2	2	1	2	1	5	ICA			5	< 1/3 MCA	no		4	5		X		X	X
8	1	62	27	2	2	1	2	2	2	1	3	ICA			5	> 1/3 MCA	no		4	1		X		X	X
9	1	66	0	2	2	1	1	2	1	2	4	ICA			0	< 1/3 MCA	no		0	1		X		X	
10	2	75	9	2	1	1	1	1	1	1	4	ICA			5	> 1/3 MCA	no		4	1		X		X	X
11	1	71	3	2	1	2	2	2	1	1	4	ICA			2a	<1/3 MCA	no		2	2		X		X	
12	1	55	8	2	2	1	2	2	1	1	5	ICA			5	< 1/3 MCA	no		4	5				X	
13	1	61	4	2	2	1	2	1	1	1	5	ICA			5	< 1/3 MCA	no		0	1				X	
14	2	75	4	2	1	1	2	1	2	1	5	ICA			0	< 1/3 MCA	no		2	1				X	X
15	1	74	2	2	1	1	2	1	2	1	5	ICA			5	< 1/3 MCA	no		1	1		X		X	
16	2	46	10	1	1	1	1	2	1	1	5	ICA			5	> 1/3 MCA	no		4	5		X		X	
17	1	77	4	2	1	2	2	1	2	1	1	ICA			5	< 1/3 MCA	no		2	2		X		X	
18	1	55	13	2	1	1	1	2	1	2	5	ICA			5	> 1/3 MCA	HT1	no	3	1		X		X	
19	2	67	3	2	1	1	2	2	1	1	5	ICA			5	< 1/3 MCA	no		3	1		X		X	X
20	1	49	6	2	1	1	2	2	1	1	4	ICA			5	< 1/3 MCA	no		4	4, dissection		X			X
21	2	83	5	2	1	2	2	1	2	1	5	ICA			5	< 1/3 MCA	no		1	2		X			
**ICA-MCA occlusion without IVT/EVT (6 Pat)**																									
22	2	88	19	2	1	2	1	1	1	1	4	ICA + M1			0	> 1/3 MCA	no		6	2		X			X
23	1	55	22	2	2	1	2	1	1	1	3	ICA + M1			5	> 1/3 MCA	no		6	5		X		X	
24	2	85	21	2	1	1	1	1	1	1	5	ICA + M1			5	> 1/3 MCA	no		6	1		X			X
25	1	69	14	2	2	1	1	1	1	2	5	ICA + M1			0	> 1/3 MCA	HT1	no	4	1		X		X	
26	1	43	11	1	1	1	2	2	1	1	4	ICA + M1			5	> 1/3 MCA	no		5	4, dissection		X			X
27	1	55	12	2	2	1	1	1	2	1	5	ICA + M1			0	> 1/3 MCA	no		4	4, dissection		X			X
**ICA occlusion IVT (10 Pat)**																									
28	2	43	18	2	2	1	2	1	1	1	1	ICA	84		0	< 1/3 MCA	no		2	4, dissection		X		X	X
29	2	76	9	2	2	1	1	1	2	1	1	ICA + M2	270		0	< 1/3 MCA	no		4	1		X		X	
30	2	84	19	2	1	2	1	1	2	1	1	ICA	85		0	> 1/3 MCA	no		4	2		X		X	
31	1	76	9	2	1	2	2	1	1	1	1	ICA	156		0	> 1/3 MCA	no		4	2		X		X	
32	2	85	12	2	1	2	2	1	1	1	1	ICA	225		0	> 1/3 MCA	no		6	2		X		X	
33	1	74	3	2	1	1	2	1	1	2	1	ICA + M2	75		0	< 1/3 MCA	no		1	1		X		X	
34	2	56	13	2	1	1	2	2	1	1	2	ICA	25		0	> 1/3 MCA	PH1	no	3	1		X			X
35	1	76	21	2	1	1	1	1	1	1	1	ICA	70		0	> 1/3 MCA	no		6	5		X		X	
36	1	76	5	2	1	1	1	1	2	1	1	ICA	90		0	< 1/3 MCA	no		0	1		X		X	
37	1	86	15	2	1	2	1	1	1	1	2	ICA	270		1	< 1/3 MCA	no		5	2				X	
**ICA occlusion IVT/EVT (12 Pat)**																									
38	1	64	17	1	1	1	1	2	1	1	2	ICA	244	330	3	>1/3 MCA	PH2	yes	4	1		X			X
39	1	58	20	2	1	1	1	1	1	1	1	ICA	75	139	3	<1/3 MCA	no		2	4, dissection		X		X	
40	1	44	2	2	2	1	2	2	2	1	1	ICA	85	329	3	<1/3 MCA	no		1	4, dissection		X		X	
41	1	69	13	2	1	1	1	1	1	1	2	ICA	90	164	2a	>1/3 MCA	HT1	no	5	5				X	
42	2	51	3	1	1	1	1	1	1	1	1	ICA + M2	224	265	3	<1/3 MCA	no		0	4		X			X
43	1	75	11	2	2	1	2	1	1	1	2	ICA	99	191	3	<1/3 MCA	no		3	1		X		X	
44	1	57	9	2	2	2	2	1	1	1	1	ICA + M2	166	259	3	>1/3 MCA	PH1	yes	6	2		X		X	
45	1	76	11	2	1	1	2	1	1	2	1	ICA	75	160	3	<1/3 MCA	no		0	2		X		X	
46	2	63	2	2	1	1	2	2	2	1	1	ICA	63	154	3	< 1/3 MCA	no		1	1		X	X	X	
47	2	62	16	1	1	1	1	1	1	1	1	ICA	68	111	3	>1/3 MCA	PH2	yes	6	5				X	
48	1	77	14	2	1	2	2	1	1	1	1	ICA	60	120	3	> 1/3 MCA	HI2	no	4	1		X			X
49	2	77	26	2	1	2	2	2	1	1	1	ICA	52	124	3	< 1/3 MCA	no		0	2		X		X	X
**ICA-MCA occlusion IVT (13 Pat)**																									
50	2	98	17	2	1	2	2	1	2	1	1	ICA + M1	130		1	> 1/3 MCA	no		6	2		X		X	
51	2	74	13	1	1	1	1	1	2	1	1	ICA + M1	120		3	> 1/3 MCA	no		5	1		X		X	
52	2	95	17	2	1	2	2	1	1	1	2	ICA + M1	115		0	> 1/3 MCA	HT1	no	6	2				X	X
53	2	93	12	2	1	2	2	1	1	1	1	ICA + M1	180		3	< 1/3 MCA	no		1	5		X		X	
54	1	52	18	2	1	1	1	2	1	1	1	ICA + Carotid T	68		1	> 1/3 MCA	no		5	1		X			X
55	1	87	17	2	1	2	2	2	1	2	1	ICA + Carotid T	75		0	> 1/3 MCA	no		6	5		X		X	
56	2	82	26	2	1	2	2	1	1	1	1	ICA + Carotid T	123		5	> 1/3 MCA	no		5	2		X		X	
57	1	64	16	2	2	2	2	1	1	2	1	ICA + Carotid T	93		0	> 1/3 MCA	no		6	5				X	
58	2	85	19	2	1	2	2	1	1	2	1	ICA + M1	105		2a	> 1/3 MCA	no		6	2		X		X	
59	1	77	9	2	1	1	2	1	1	2	1	ICA + M1	109		3	> 1/3 MCA	no		5	1		X		X	
60	1	87	18	2	1	1	1	1	1	1	2	ICA + M1	190		5	> 1/3 MCA	PH1	yes	6	5		X		X	X
61	2	86	23	1	1	2	1	1	2	1	2	ICA + M1	125		5	> 1/3 MCA	no		6	1		X		X	
62	2	89	16	2	1	2	1	1	1	1	1	ICA + M1	110		0	> 1/3 MCA	no		6	2		X		X	
**ICA-MCA occlusion IVT/EVT (16 Pat)**																									
63	1	86	15	2	1	1	1	1	1	1	2	ICA + Carotid T	90	120	2b	< 1/3 MCA	no		2	1		X			X
64	1	77	15	1	1	1	1	1	1	1	1	ICA + M1	33	74	2b	> 1/3 MCA	HT1	no	3	1		X		X	
65	2	81	16	2	1	2	1	1	1	1	1	ICA + Carotid T	32	195	3	> 1/3 MCA	no		6	2				X	
66	1	69	15	1	1	2	1	1	1	1	2	ICA + Carotid T	260	330	2a	< 1/3 MCA	no		5	2		X		X	
67	2	61	21	2	1	1	2	2	1	1	2	ICA + Carotid T	85	176	0	< 1/3 MCA	PH2	yes	3	5		X			
68	2	71	5	2	1	1	2	1	1	1	1	ICA + Carotid T	150	206	3	> 1/3 MCA	PH2	yes	6	5		X		X	
69	2	51	18	2	2	1	2	1	1	1	1	ICA + Carotid T	105	251	2a	> 1/3 MCA	PH2	yes	6	2				X	
70	2	75	25	2	1	2	1	1	1	1	1	ICA + M1	125	245	2a	> 1/3 MCA	HT1	yes	3	2		X		X	
71	1	73	20	3	1	2	1	1	1	2	1	ICA + Carotid T	120	177	3	> 1/3 MCA	PH1	no	6	2				X	
72	2	70	13	2	1	1	1	1	1	1	2	ICA + M1	45	101	3	> 1/3 MCA	PH1	yes	6	1		X		X	
73	2	84	9	2	1	2	2	1	1	1	1	ICA + M1	210	260	0	< 1/3 MCA	no		5	2		X		X	
74	1	52	14	1	1	1	1	2	1	1	1	ICA + M1	70	139	3	< 1/3 MCA	PH1	no	0	1		X		X	
75	1	55	10	2	1	1	2	2	1	1	1	ICA + Carotid T	20	80	3	< 1/3 MCA	PH1	no	3	1		X		X	
76	2	55	10	2	1	1	2	1	1	1	2	ICA + M1	225	416	3	< 1/3 MCA	PH1	no	2	1		X		X	
77	2	73	16	1	1	1	2	1	1	1	2	ICA + M1	51	167	2a	> 1/3 MCA	no		3	1		X		X	
78	1	77	16	2	1	1	2	1	1	1	1	ICA + M1	87	155	3	< 1/3 MCA	no		2	5		X		X	
